# Integrative Transcriptome and Proteome Analysis Identifies Major Molecular Regulation Pathways Involved in Ramie (*Boehmeria nivea* (L.) Gaudich) under Nitrogen and Water Co-Limitation

**DOI:** 10.3390/plants9101267

**Published:** 2020-09-25

**Authors:** Jikang Chen, Gang Gao, Ping Chen, Kunmei Chen, Xiaofei Wang, Lianyang Bai, Chunming Yu, Aiguo Zhu

**Affiliations:** 1 Longping Branch, Graduate School of Hunan University, Changsha 410082, China; chenjikang@caas.cn; 2 Institute of Bast Fiber Crops, Chinese Academy of Agricultural Sciences, Changsha 410205, China; gaogang@caas.cn (G.G.); chenping02@caas.cn (P.C.); chenkunmei@caas.cn (K.C.); wangxiaofei@caas.cn (X.W.); 3 National Breeding Center for Bast Fiber Crops, Changsha 410125, China; 4 Hunan Academy of Agricultural Sciences, Changsha 410125, China

**Keywords:** ramie (*Boehmeria nivea* (L.) Gaudich), nitrogen and water co-limitation, transcriptome, proteome, synergistic effects

## Abstract

Water and N are the most important factors affecting ramie (*Boehmeria nivea* (L.) Gaudich) growth. In this study, de novo transcriptome assembly and Tandem Mass Tags (TMT) based quantitative proteome analysis of ramie under nitrogen and water co-limitation conditions were performed, and exposed to treatments, including drought and N-deficit (WdNd), proper water but N-deficit (WNd), proper N but drought (WdN), and proper N and water (CK), respectively. A total of 64,848 unigenes (41.92% of total unigenes) were annotated in at least one database, including NCBI non-redundant protein sequences (Nr), Swiss-Prot, Protein family (Pfam), Gene Ontology (GO) and KEGG Orthology (KO), and 4268 protein groups were identified. Most significant changes in transcript levels happened under water-limited conditions, but most significant changes in protein level happened under water-limited conditions only with proper N. Poor correlation between differentially expressed genes (DEGs) and differentially expressed proteins (DEPs) was observed in ramie responding to the treatments. DEG/DEP regulation patterns related to major metabolic processes responding to water and N deficiency were analyzed, including photosynthesis, ethylene responding, glycolysis, and nitrogen metabolism. Moreover, 41 DEGs and 61 DEPs involved in regulating adaptation of ramie under water and N stresses were provided in the study, including DEGs/DEPs related to UDP—glucuronosyhransferase (UGT), ATP synthase, and carbonate dehydratase. The strong dependency of N-response of ramie on water conditions at the gene and protein levels was highlighted. Advices for simultaneously improving water and N efficiency in ramie were also provided, especially in breeding N efficient varieties with drought resistance. This study provided extensive new information on the transcriptome, proteome, their correlation, and diversification in ramie responding to water and N co-limitation.

## 1. Introduction

Global yield variability is heavily controlled by nitrogen application and irrigation [1]. Profoundly negative environmental effects of increased concentrations of reactive N in the biosphere, and even greater consumption of fresh water for supporting agriculture have led researchers to call into question the feasibility of enhancing nitrogen- and water-use efficiency simultaneously [2,3]. Previous studies have identified agricultural systems with strong interactions between water- and nitrogen-use efficiency, and suggested that management practices oriented toward reducing nitrogen losses and maintaining farm productivity should rely on optimizing nitrogen and water inputs at the same time [3]. One solution to this issue is production of crop varieties that are highly efficient in using nitrogen/water and produce high yields with reduced nitrogen/water input [4,5,6]. Generally, both water and nitrogen addition increase plant biomass production [7]. However, the addition of water and nitrogen can have additive [8], non-additive [9], amplifying or even antagonistic effects [10]. These effects are species specific, which indicates different physiological mechanism of different crops responding to different water–nitrogen conditions. Therefore, better understanding of physiological mechanisms of specific crops induced by nitrogen-water co-limitation is important for genetic improvement of crop varieties.

Ramie (*Boehmeria nivea* L.) is widely cultivated as a perennial herb, mainly in Asian countries, and has been used as a fiber crop for over 4000 years [11]. The fiber obtained from ramie is known as the longest and one of the strongest fine textile fibers [12]. Besides, ramie is also used as medicine [13], forage [14], phytoremediation plants [15], biochar materials [16], and mushroom substrate [17]. Although ramie has a wide adaptation capacity, the plant construction and yield are significantly influenced by nutrient and water supply [18,19]. Usually, soil nitrogen and water are the most important factors affecting the growth of ramie and large amounts of N and water are required. Up to 280 kg/hm^2^ of N would be used to maximize ramie yield [20] and the fiber yield could be decreased by more than 30% under N-deficit condition [21]. Compared to well-watered plants, the fiber yield, stem length, diameter, and bark thickness of ramie were decreased by 26.7%, 23.5%, 17.7%, and 19.7% under drought stress [18,19]. In recent years, along with the shifting of ramie production from plain lake areas to rainfed uplands in China, farmers are forced to face greater intense droughts and nitrogen limitation/losses. There is a pressing need to improve nitrogen- and water-use efficiency of ramie. However, to our knowledge, only few studies have discussed plant response to single factor, but none was involved in nitrogen and water co-limitation.

Strong stress resistance and/or compensatory growth capacity in plants will ensure survival and reduce production losses [22], which gives rise to the necessity of understanding the molecular regulation mechanism of plants. A number of studies have reported molecular responses to nitrogen- or water-deficit and responses to nitrogen re-supply or rewatering, including grain crops, such as *Oryza sativa* [23], fruit trees, such as *Pyrus bretschneideri* [24], and oilseed crops, such as *Sesamum indicum* [25]. Unfortunately, little was known about nitrogen–water co-limitation. As a perennial crop, the subsequent growth and yield of ramie is affected by the seedling morphological construction and growth vigor significantly within a long (or even the whole) growth period. Thus, it is important to study the underlying molecular mechanism of ramie seedling responding to individual and combined water and N limitation.

Previous studies have shown that the combination of proteome and multiple histology can be used to study the physiological and biochemical changes induced by abiotic stress. Therefore, combined transcriptome and proteome analyses were conducted to investigate the global transcriptome and proteome profiles of ramie under water–nitrogen co-limitation conditions to gain a broader systematic view of ramie adaptation to the combined stresses, and to identify additional common (as well as distinct) molecular regulatory events under different stress conditions. This study will provide a detailed framework of leaf proteome profiles and the association and difference with transcriptome profiles under different stress conditions.

## 2. Materials and Methods

### 2.1. Plant Growth and Sampling

Ramie (*Boehmeria nivea* L.) variety, Zhongzhu No. 2, was provided by Institute of Bast Fiber Crops (Changsha, China). Cuttings of the variety, 13.5 ± 1.5 cm in length and 4.5 ± 0.5 g in weight, were collected and rooted in hydroponic apparatus only with water in a plant growth chamber in July 2019. The plants were grown in a greenhouse, in which the relative humidity was 60%, temperature was 30 °C, and the photoperiod was 16 h/8 h (light/dark). After 2 weeks, ramie plants were transplanted in potted soil and each plant was grown in one pot as a replicate. After 10 days of culturing only by water, they were divided into four groups with six biological replicates in each and exposed to treatments including drought and N-deficit (WdNd), proper water but N-deficit (WNd), proper N but drought (WdN), and proper N and water (CK), respectively. The composition of the nutrient solutions was showed in Table 1. The WdNd and WdN treatments were cultured by 50 mL/d of the corresponding nutrient solutions without additional water. The WNd and CK treatments were cultured by 100 mL/d of the corresponding nutrient solutions with additional water to keep the soil water content maintained at 80% of soil field capacity. After 2 weeks of growth, the plant height and leaf numbers were measured and the third to fourth fully expanded leaves were collected for omics analysis. Sample from two plants were merged together as one biological replicate and three biological replicates were used for omics analysis.

### 2.2. RNA Sample Preparation and Transcriptome Analyses

Total RNA was extracted using TRIzol Reagent (Life Technologies, Carlsbad, CA, USA) following the manufacturer’s instructions, and the RNA integrity was verified using an Agilent 2100 RNA Nano 6000 Assay Kit (Agilent Technologies, Santa Clara, CA, USA). Methods S-1 provided details on RNA-Seq libraries construction, sequencing and bioinformatics analysis [26].

### 2.3. Protein Sample Preparation and Proteomic Analysis

The procedure for protein sample preparation and proteomic analysis mainly included proteins extraction, reduction, alkylation, trypsin digestion, Tandem Mass Tags (TMT) labeling of peptides, liquid chromatography linked to tandem mass spectrometry (LC-MS/MS) (Applied Protein Technology, Shanghai, China), protein identification, and quantitation, and bioinformatic analysis (Gene Ontology (GO), KEGG Orthology (KO)). Details were shown in Methods S-2 [27,28,29]. The protein-protein interaction network (PPI) analyses were conducted using Cytoscape 3.X to predict the effect of water and N stress on the protein functioning pattern in ramie. The PPI connectivity degree represented the number of differentially expressed proteins (DEPs) interacted with the given DEP.

### 2.4. Association Analysis of Transcriptome and Proteome Profiles

The number of clean reads for each gene was calculated and normalized to reads per kilobase per million reads (RPKM) for gene expression analysis. Genes with a false discovery rate (FDR) *p_adj_* < 0.05 and |log2Foldchange| ≥ 1 were designated as differentially expressed genes (DEGs). Proteins with a *p_adj_* < 0.05 and fold change > 1.2 or <0.83 were designated as DEPs. Otherwise, the genes were designated as non-differentially expressed genes (NDEGs) and the proteins were designated as non-differentially expressed proteins (NDEPs). A gene and its corresponding protein were considered to be correlated in compared treatments if both the gene and protein were expressed in each treatment. According to the expressing trend of proteins and genes, Pearson correlated coefficient of five different correlated types, including DEPs/DEGs-SameTrend, DEPs/DEGs-Opposite, DEPs/NDEGs, NDEPs/DEGs, and NDEPs/NDEGs, were calculated [30,31,32,33]. DEPs/DEGs-SameTrend indicated same trend in differentially expressed proteins and mRNAs, such as upregulated DEPs simultaneously with upregulated DEGs, or downregulated DEPs simultaneously with downregulated DEGs. DEPs/DEGs-Opposite indicated opposite trend in DEPs and DEGs, such as upregulated DEPs simultaneously with downregulated DEGs and vice versa [31]. DEPs/NDEGs indicated that there were DEPs but non-correlated DEGs. NDEPs/DEGs indicated that there were DEGs but non-correlated DEPs. NDEPs/NDEGs indicated that there were none DEPs or DEGs. Cluster analysis was used to identify groups of similarly differentially expressed proteins and transcripts with Cluster 3.0.

### 2.5. Statistical Analysis

Plant phenotypic data, including plant height and fully expanded leaf numbers, was tested by the Student-Newman-Keuls (SNK) method with IBM Statistics SPSS 19.0 (*p* < 0.05).

## 3. Results

### 3.1. Seedling Morphological Differences among Different Nitrogen–Water Conditions

**Figure 1:**
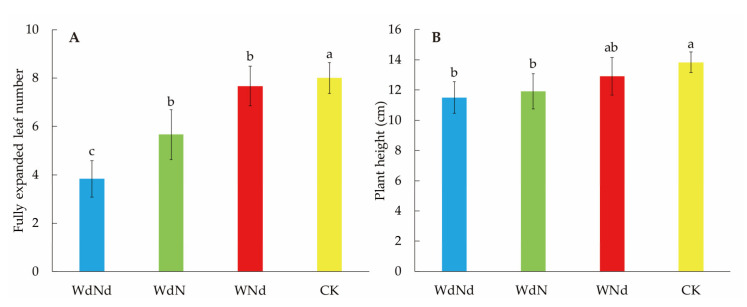
Fully expanded leaf numbers (**A**) and plant height (**B**) of ramie seedlings under different nitrogen-water conditions. The error bar represents the standard error. The different letters in the chart indicate significant differences at *p* < 0.05 among treatments according to Student-Newman-Keuls (SNK) test. WdNd presents the treatment with drought and N-deficit. WNd presents the treatment with proper water but N-deficit. WdN presents the treatment with proper N but drought. CK presents the treatment with proper N and water. The same as below.

Ramie seedling morphology was sharply shaped by different nitrogen–water conditions (Figure 1). The growth of ramie was significantly decreased under water and N-deficit (WdNd) compared to proper conditions (CK) according to expanded leaf numbers and plant height. Water-deficit (WdN) hindered ramie seedling growth worse than only nitrogen-deficit treatment (WNd), which indicated that water was the primary factor affecting ramie seedling growth. More leaves and higher plant under WdN than WdNd indicated that N application might mitigate the impact of water-deficit on ramie growth.

### 3.2. Overview of Ramie Transcriptome and Proteome

A total of 42,862,674 to 83,799,500 raw reads were obtained from the 12 sequencings (3 biological replicates were performed for each treatment) in which 40,272,97–883,365,184 high-quality clean reads were obtained with Q20 percentage over 97% and a GC percentage ranged from 49.01–52.59%. After de novo assembling using Trinity, functional annotation was conducted against five public databases (Table 2). A total of 64,848 unigenes (41.92%) were annotated in at least one database, including NCBI non-redundant protein sequences (Nr) (56,802, 36.72%), Swiss-Prot (35,660, 23.05%), Protein family (Pfam) (39,358, 25.44%), GO (26,711, 17.27%) and KO (11,912, 7.7%).

GO analysis showed that cellular process and metabolic process were dominant in “biological process” category, catalytic activity and binding were dominant in “molecular function” category, and cell, cell junction and membrane were dominant in “cellular component” category (Supplementary Figure S1).

The KEGG annotated unigenes were classified into 5 main categories with 34 subcategories (Supplementary Figure S2). The results indicated that pathways involved in transport and catabolism (1018, 8.55%), cell growth and death (932, 7.82%), signal transduction (2078, 17.44%), translation (1156, 9.70%), folding, sorting and degradation (821, 6.89%), global and overview maps (6514, 54.68%), carbohydrate metabolism (1311, 11.01%), amino acid metabolism (953, 8.00%), and immune system (691, 5.80%) were most active in ramie responding to nitrogen and water changes.

**Figure 2:**
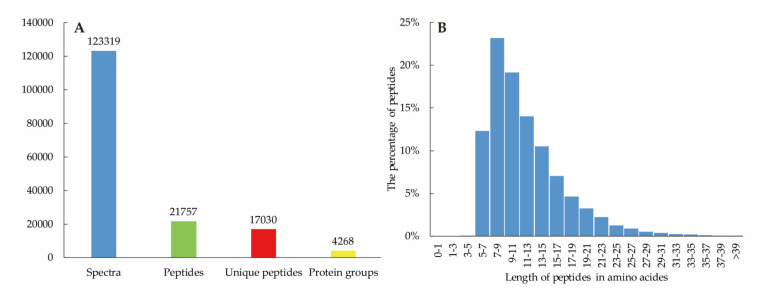
Number of proteins identified (**A**) and the length distribution of peptides (**B**) in ramie under different water and N conditions.

Totally, 123,319 spectra were acquired, 21,757 peptides (17,030 unique peptides) were detected and 4268 proteins were identified (Figure 2). The lengths of peptides in amino acids were mainly distributed from 521—which suggesting that sampling reached the standard required. The details of all identified proteins were shown in Supplementary Table S1.

### 3.3. Identified DEGs and DEPs Involved in Ramie Nitrogen and Water Stress Responses

**Figure 3:**
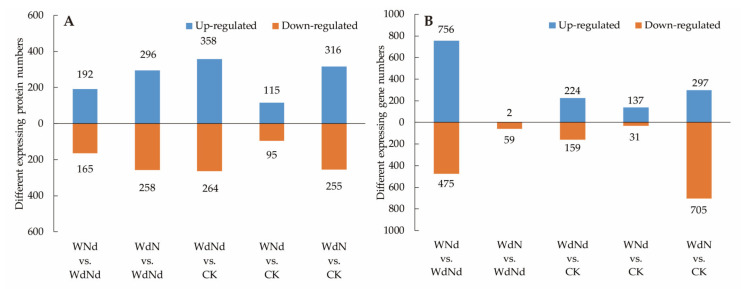
Differentially expressed genes (**A**) and differentially expressed proteins (**B**) according to different water and nitrogen treatments.

The distribution of DEGs among treatments were showed in Figure 3. The DEG numbers of the treatments with same N condition but different water conditions (2233, including WNd vs. WdNd and WdN vs. CK) were larger than the DEG numbers of the treatments with same water condition but different N conditions (229, including WdN vs. WdNd and WNd vs. CK). It was interesting that the co-limitation of water and N (WdNd) causes less gene expressing changes compared with single water changes but more compared with single N changes. The result indicated that the different gene expression patterns of ramie were mainly induced by water in water–nitrogen interaction and synchronizing water- and N-stresses might be a way for saving N.

There were only slight differences between the amounts of upregulated DEGs (1416) and downregulated DEGs (1429) along all treatments, but obvious differences were showed in each comparing group. For example, the number of upregulated genes compared with WNd and WdNd was 756, which was 1.59 times more than the number of downregulated genes; in contrast, the number of upregulated genes compared with WdN and CK was 297, which was only 42% of the number of downregulated genes.

The numbers of DEGs in different comparison were significantly correlated to the numbers of DEPs according to Pearson test (*p* < 0.05, *r* = 0.695). Like DEGs, the DEP numbers (928) of the treatments with the same N condition, but different water conditions were also larger than the DEP numbers (764) of the treatments with the same water condition, but different N conditions. Moreover, there were slight differences between the amounts of total upregulated DEPs (1277) and downregulated DEPs (1037) along all treatments (Figure 3). However, the expressing patterns of DEPs were utterly different from DEGs. The distribution of up- and downregulated DEPs were more evenly compared to DEGs. The DEP number of WdNd vs. CK was the highest compared to other groups. More DEPs were detected in WdN than in WNd when comparing to WdNd or CK, but the same trend of DEGs was only detected when compared to CK. It implied that application of N under the WdNd condition regulated the plant growth, mainly at protein rather than the gene expression level. The gene expression of ramie was more sensitive to application of water than proteins under WdNd condition. On the other hand, compared to proper water and N conditions (CK), both DEGs and DEPs were more sensitive to water-deficit than N-deficit.

Four major metabolic processes were investigated in order to profile the gene regulation pattern responding to different water and N conditions (Table 3). As the number of up- and downregulated DEGs showed, more DEGs, especially upregulated DEGs, were induced under proper water conditions with or without enough N. The N addition induced more downregulated than upregulated DEGs.

**Figure 4:**
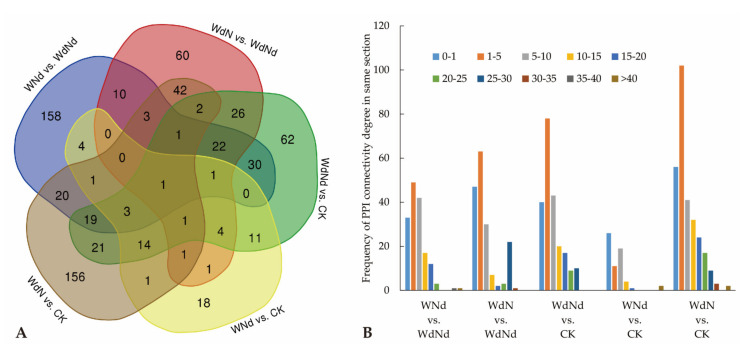
Protein to protein interactive network (PPI) of DEPs responding to different water and N conditions. (**A**) Showed the Venn diagram of interactive DEP numbers. (**B**) Showed the PPI connectivity degree distribution of DEPs responding to different water and N conditions.

As results show in Figure 4A, the PPI differentiated in at least three levels along comparisons: (1) total number of interactive DEPs, (2) the number of common and unique interactive DEPs among comparisons, and (3) different common interactive DEPs. The PPI connectivity degree presented different patterns along comparisons at the aspects of average value, highest value, and distribution (Figure 4B). As the only DEP interacting with others, TRINITY_DN29638_c0_g1 was identified as a protein, which produces ATP from ADP in the presence of a proton gradient across the membrane.

### 3.4. Correlation of Transcript and Protein Profiles in Ramie under Water and Nitrogen Stress

**Figure 5:**
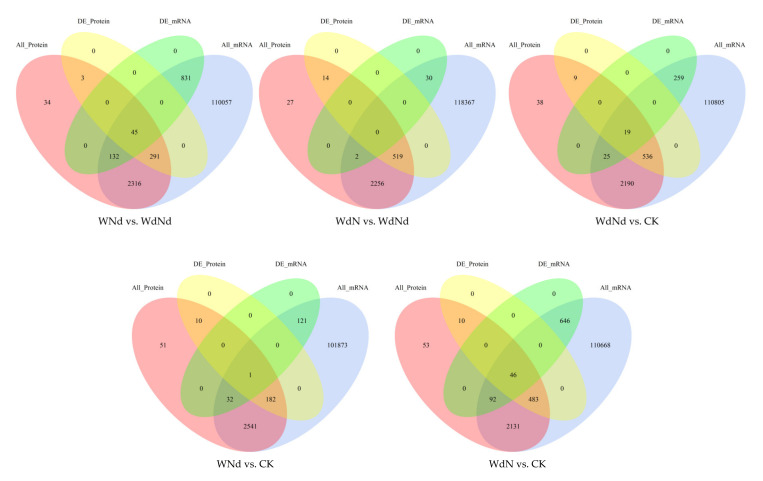
Correlated genes and proteins expressed in ramie under different water and N conditions. All_protein/mRNA present the numbers of total proteins/mRNAs identified in the treatments. DE_ protein/mRNA present the numbers of differentially expressed proteins/mRNAs identified in the treatments. The numbers in the overlapped parts of the ellipses were the numbers of correlated proteins and/or mRNAs.

The correlations of DEGs and DEPs involved in ramie responses under different water and nitrogen stress were analyzed (Figure 5). A small part of DEGs were correlated to DEPs according to all comparisons. Up to 6.27% of DEGs were detected to correlate with DEPs along all the treatments. Most DEGs were detected without correlated proteins, but most DEPs were correlated with mRNAs.

Concerning the WNd vs. WdNd (45) and the WdN vs. CK (46), comparisons differentiated in water conditions, but with the same N conditions, no matter the deficit or proper, and had the most correlated differentially expressed factors. On the contrary, with the WdN vs. WdNd and WNd vs. CK, comparisons with same water conditions, but differentiated in N conditions, had the least correlated differentially expressed factors. The number of correlated differentially expressed factors of ramie under combined stress was in between.

According to the expressing trend of proteins and genes, coefficient of five different correlated types were calculated (Table 4). Generally, same expressing trends were detected in most correlated DEPs/DEGs. It was interesting that the DEP expressing level was negatively correlated to DEG in WdNd vs. CK, although they had a same expressing trend. The coefficient was low along all the comparisons and correlated types, which indicated a complex regulation mechanism of ramie responding to water and N stress. As the cluster analysis showed, ramie represented a different expressing pattern between genes and proteins under different water and N conditions (Supplementary Figure S3–S5).

### 3.5. GO Enrichment Analysis Based on DEPs Correlated to DEGs

**Figure 6:**
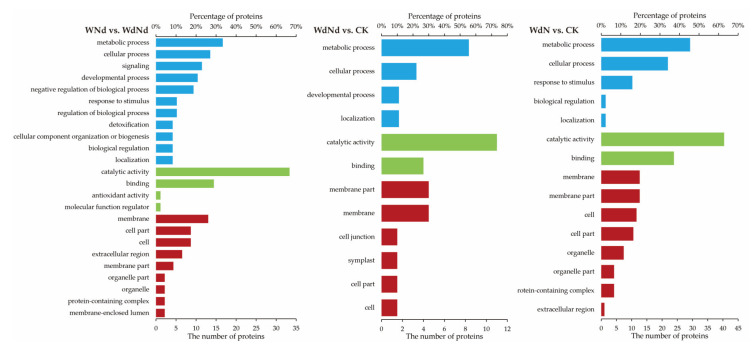
GO level 2 terms (Correlated *P* value < 0.05) for the DEPs associated to DEGs. The blue, green, and red terms present biological process (BP), molecular function (MF), and the cellular component (CC) category, according to GO analysis, respectively.

Only three comparison groups were taken for GO enrichment analysis based on DEPs associated to DEGs for none (or only one) correlated factor, and was detected in the other two groups (Figure 6). The result implied that drought induced more complete metabolic pathways than N-deficit. The GO terms were mainly enriched in the metabolic process, and cellular process belonged to the biological process (BP) category, in catalytic activity, and binding belonged to the molecular function (MF) category, and membrane, membrane part, cell, and cell part belonged to the cellular component (CC) category. GO terms, belonging to BP, and enriched in comparisons with individual water difference was contrasted with the combined stress treatment (WdNd vs. CK). A similar performance was detected in the CC category. The results indicated that interaction effects of water and N should change the molecular responding profile of ramie, especially in the developmental process, response to stimulus, extracellular region, and organelle.

### 3.6. KEGG Pathway Enrichment Analysis Based on Transcriptome and Proteome

The KEGG analysis showed that four pathways were enriched in WNd vs. WdNd, including amino sugar and nucleotide sugar metabolism, pentose, and glucuronate interconversions, metabolism of xenobiotics by cytochrome P450, and drug metabolism—cytochrome P450 (Table 5). Although correlated DEPs/DEGs were enriched into different KEGG pathways, according to comparisons, the pathways were mainly related to photosynthesis, glycolytic, and the immune system.

## 4. Discussion

### 4.1. Ramie Growth under Different Water and N Conditions

In the present study, the integrative transcriptome and proteome analysis method was used to investigate the systematic view of regulation mechanism of ramie under combined water and N stresses. Four treatments with different water and N conditions were conducted and five groups were compared to insight the molecular responding profiles. WdN vs. WdNd could conduce to reveal the effects of N application on the ramie molecular profile under the co-limitation condition. WNd vs. WdNd was helpful for revealing the effects of water application on ramie under the co-limitation condition. WdNd vs. CK could be conducive to reveal the effects of co-limitation on ramie. WdN vs. CK contributed to reveal the effects of water-deficit on ramie. WNd vs. CK was useful for understanding the effects of N-deficit on ramie. Moreover, the comparison among groups was helpful for identifying the differences of molecular profiles according to single or combined stresses. As we know, it was the first time that the molecular regulation mechanism of ramie under water and N co-limitation was discussed.

Water and/or N deficiency restricted ramie growth significantly, according to the less fully expanded leaf number and lower plant height (Figure 1). Ramie growth was more sensitive to water-deficit than N-deficit and the combined stress restricted the plant worst. As a substantial reduction in the number of fully expanded leaf was detected, the plant photosynthesis and biomass accumulation were hindered. On the other hand, applying water or N to the combined stress condition enhanced the plant growth according to the results comparing WdN and WNd to WdNd. As water limitation could lead to crop nitrogen deficit [34], weaker plant growth was observed in WdN than in WNd. Applying N alleviated the adverse effects under the combined stress, which was consistent with previous studies [9,35].

### 4.2. Photosynthesis and Ethylene Responding in Ramie under Water and N Stresses

Leaf senescence affects plant production crucially by photosynthetic impairment [36]. Poor fiber and biomass production resulting from leaf senescence and leaf abscission is a significant problem in ramie [37]. In the present study, the remaining leaves and plant height of ramie under stressed conditions were significantly decreased compared to CK (Figure 1). The result implied that the molecular regulation on leaf senescence should play important roles in ramie responding to water and N stresses. As a key hormonal control, ethylene stimulates plant senescence [38] affects synthesis and recovery of antioxidants [39] and, consequently, restricts leaf expansion or even leads to wither. Ethylene responding genes might regulate ethylene production positively or negatively. Thus, they affect leaf senescence in both directions. Similar regulation patterns were detected in DEG/DEP related photosynthesis and ethylene responding (Table 5). Water application induced more photosynthesis-related DEGs/DEPs than N addition no matter under combined water and N stress or individual drought stress.the N conditions (WNd vs. WdNd, WdN vs. CK). However, ethylene production and photosynthesis were opposites regarding plant growth. The result suggested that DEGs/DEPs related to ethylene responding regulated ramie senescence negatively under deficient water and N conditions. On the contrary, DEGs/DEPs related to photosynthesis regulated ramie growth positively. Although the DEGs and DEPs represented similar regulation trend, there was a large gap between the number of DEGs and DEPs. The results also indicated the advantages of combined analysis of transcriptome and proteome in better understanding of physiological and biochemical changes of various biological processes [40].

### 4.3. Glycolysis in Ramie under Water and N Stresses

Energy deprivation, caused by reduced photosynthesis or leaf senescence, is a general symptom of stressed photosynthetic plants [41]. Often, plants induce glycolysis to maintain inherent energy balance under stressed environment [42]. Deng et al. indicated that ramie responded to N stress by enhancing secondary metabolism and reducing photosynthesis and energy metabolism to increase endurance, especially by increasing signal transduction pathways, enhancing the connection between glycolysis and photosynthesis, promoting the intracellular flow of carbon and N [43]. In the present study, numbers of DEGs related to glycolysis were upregulated under water and/or N deficiency compared to proper conditions (Table 3, WdNd vs. CK, WNd vs. CK, WdN vs. CK). DEGs and DEPs correlated significantly in glycolysis pathways, especially in WNd vs. WdNd (Table 5). Water addition (WNd) induced 54 upregulated and 21 downregulated DEGs compared to WdNd (details in Supplementary Table S2). Only one downregulated DEG, annotated as UDP-glucosyltransferase (UGT), were detected in WdN compared to WdNd. The results indicated that water was the main driven force for regulating ramie glycolysis, and the only downregulated DEG (TRINITY_DN97302_c2_g1) in WdN vs. WdNd might play an important role in responding to N addition under combined water and N stress condition. Our previous study had showed differences in nitrogen use efficiency (NUE) between genotypes and clarified the importance of UGT related genes in high-NUE variety [44]. While plant cells contain a large number of UGT isozymes that have a wide range of functions [45], it gives a new sight for further study in water and N interactions. Meanwhile, according to the PPI analysis, great concern should be paid to the common interactive protein TRINITY_DN29638_c0_g1, identified as ATP synthase subunit beta producing ATP from ADP in the presence of a proton gradient across the membrane.

### 4.4. Nitrogen Metabolism in Ramie under Water and N Stresses

**Figure 7:**
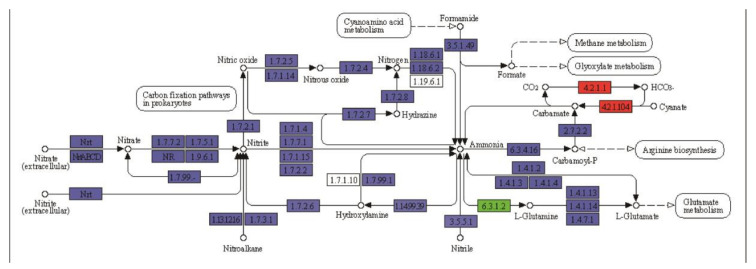
KEGG map of differentially expressed proteins enriched in nitrogen metabolism in WdN vs. WdNd. Colored square columns indicate identified proteins in the experiment, in which red columns indicate the proteins upregulated responding to the treatment, and the green column in the figure indicates the protein downregulated responding to the treatment.

It was predicted that N assimilation would be enhanced as a method of supplying N demands under N stress condition [46]. The role of some key transcription factors, including MYB ((v-myb avian myeloblastosis viral oncogene homolog), AP2/ERF (APETALA2/ethylene responsive factor), bZIP (basic leucine zipper), *Dof* (DNA Binding with one finger), etc., involved in drought or N-deficit stress responses, has been identified in ramie [20,47,48,49]. Previous studies also clarified the role of some genes involved in drought/N-deficit responses in ramie, including sucrose synthase genes, phloem protein genes, and glutamine synthetase gens [50,51,52]. However, little was known in synergistic effects of water and N co-limitation. In this study, eight upregulated DEGs were detected in ramie under N-deficit condition combined with drought (Table 3, WdN vs. WdNd). Six proteins associated with N metabolism were altered. The level of glutamine synthase (TRINITY_DN102805_c3_g3), carbonate dehydratase (TRINITY_DN103396_c1_g2), nitronate monooxygenase (TRINITY_DN24063_c0_g1), and lyase (TRINITY_DN30449_c0_g1) were downregulated under N-deficit condition. It is interesting that DEP enriched in nitrogen metabolism among all the treatments included E.C. 4.2.1.1 (Figure 7). Carbonate dehydratase (carbonic anhydrase) plays a multifunctional regulatory role in all photosynthetic organisms [53]. The preventive carbonate dehydratase outcome might be due to the effects on improvement of photosynthetic capacity [54]. The result highlighted the importance of carbon–nitrogen–water interactions in regulating ramie under the combined stresses.

### 4.5. Global View on Water and N Interactions in Ramie at Molecular Level

Crop responses to the combination of water and N supply is important for evaluating and optimizing the interactions among crop management, species, cultivar, and environment [55]. Methods to investigate the mechanism of the interactions abound, but the mechanisms are mainly considered external. While soil-based methods focus on the availability of water or N, crop-based methods integrate soil availability and atmospheric demand with other drivers of crop growth and development. Little is known at the basic physiological level. In fact, plants have evolved various molecular mechanisms to reduce their consumption of resources and adjust their growth to adapt to adverse environmental conditions. Their responses are mediated by kinds of protein, e.g., plant growth regulators, compounds derived from plant biosynthetic pathways [56]. A number of studies have reported responses to nitrogen- or water-deficit on transcriptional level. Although substantial progress has been made on understanding the transcriptome dynamic of crops under nitrogen or water stress, the dynamic changes or biochemical regulation of proteins still remain largely unexplored.

Plant growth inhibition, senescence, inherent energy imbalance, and low efficiency in nutrient utilization had the most significant manifestation under combined drought and N-deficit stress. Therefore, the molecular responding characteristics related to photosynthesis, ethylene responding, glycolysis, and N metabolism were discussed to reveal the global view of regulation mechanisms of ramie under different water and N conditions. Generally, similar phenomena were detected in DEGs/DEPs according to effects of single factors, such as enhanced glycolysis, which was consistent with published research. However, based on the integrative transcriptome and proteome analysis on five comparisons with four different water and N conditions (WdNd, WdN, WNd, CK), a unique mechanism of ramie was revealed in the present study.

Ramie generally needs a high amount of N fertilizer to ensure high yields [19,57]. N was always considered to be the most sensitive nutrient factor for ramie production. Previous studies focused on the effects of low-N stress at proper moisture condition. However, ramie production was usually affected by water and N co-limitation due to the inefficiency of N application under drought and the restricted mitigation of soil inherent N. Our study revealed the strong dependency of N-responding of ramie on water conditions at the gene and protein levels. The DEG/DEP profiles differentiated significantly when N-deprived under proper water conditions (WNd vs.CK) compared to N-addition under drought conditions (WdN vs. WdNd). Moreover, different N and water conditions induced significant differentiation in molecular regulation patterns in ramie, although there were similar enriched pathways. The results emphasized the importance of water and N interactions in ramie growth. The two elements interacted through the alteration of gene/protein expressing profiles, protein-protein interaction network, the correlation of genes, and proteins and major metabolism pathways.

Plant N acquisition relies on roots and the study of genetic control of roots under nutrient limitation conditions has received great attention [58]. However, the nutrient utilization and biomass production mainly occurred in shoots, especially in leaves [59]. Moreover, the nutrient and energy exchanges between roots and shoots rely on the biological processes in shoots [60]. Acquisition and transformation phases determined the amount and location of nutrients, but the utilization phase determined the efficiency. Thus, our study was contributed to understand the physiological responses of ramie under different water and N conditions at utilization phase. We suggested that the water and N acquisition and transformation phases should be taken into consideration in further studies.

### 4.6. Advice for Improving Water and N Synergistic Effects in Ramie

Although individual water or N applications would increase ramie growth, N addition without appropriate irrigation or irrigation without appropriate N is inefficient compared to the unlimited conditions. Enhancement of water and N use efficiency simultaneously provides advantages over optimization of water and N inputs separately [3]. The improvement of water and N application in farming system is based on the optimization of interaction mechanism of the two factors. Our study has indicated that water is predominant in water and N interaction as discussed above, the priority of regulating water status should be taken with a high attention. According to the present fertilization regime of ramie, a high level of N is applied at the early stage of each harvest season. Generally, instant soluble nitrogen is applied after raining or irrigation. However, little can be done at the remaining time during ramie growth leading to a low water and N synergistic effect. We suggest that slow and precise releasing nitrogen fertilizers [61] should be used for a more precision ramie production, and integrative water and N regimes are essential for achieving higher and more efficient production [62].

Throughout the history of agriculture, crop yield can be improved with genomic-based methods by breeding cultivars to take advantage of greater resources [63]. Tolerance to high fertilizer has been a major trait in ramie breeding by far. Selection of varieties adapting to water and N limited environments has not been considered thoroughly. However, water and N co-limitation becomes a broader phenomenon under global climate change. There is an urgent need to improve varieties targeting adaptation to water and N stress simultaneously [64]. While N shortages could be overcome more easily and more cost-efficient by commercial N fertilizer than drought, N efficient cultivars with drought resistance are prioritized. Correlated DEGs/DEPs were provided in the present study, which might be effective candidates for genomic modification of ramie. The common DEGs/DEPs among all of the treatments, such as carbonate dehydratase, should be a priority of high concern. Meanwhile, the regulation trends of DEGs/DEPs should be considered for their unique regulation patterns in different metabolic pathways.

## 5. Conclusions

Ramie growth was significantly decreased by drought, N-deficit, and combined stresses. Combined transcriptome and proteome analysis technology were used for identifying molecular responses in ramie under four different water and nitrogen conditions. To our knowledge, this is the first study applying the multi-omics method to study ramie under combined water and N stresses. Different water and N conditions affected the ramie gene and protein profiles, significantly leading to a complex interaction between the two factors. However, strong dependency of N-response of ramie on water conditions at the gene and protein levels was detected. The interaction induced alterations in gene and protein expressing, especially in photosynthesis, ethylene responding, glycolysis, and N metabolism pathways. DEGs/DEPs related to UGT, ATP synthase, and carbonate dehydratase were key factors involved in regulating adaptation of ramie under water and N stresses. Advice for improving water and N synergistic effects in ramie were also provided, including the application of slow and precise releasing nitrogen fertilizers, integrative water and N regimes, and improving varieties targeting adaptation to water and N stress simultaneously. Breeding N efficient cultivars with drought resistance is prioritized.

## Figures and Tables

**Table 1:** The composition of the nutrient solutions of the treatments. WdNd presents the treatment with drought and N-deficit. WNd presents the treatment with proper water but N-deficit. WdN presents the treatment with proper N but drought. CK presents the treatment with proper N and water. The same as below.

Treatments	WdNd	WNd	WdN	CK
Ca(NO_3_)_2_·4(H_2_O)	-	-	1653.1	826.5
KNO_3_	-	-	404.4	202.2
KCl	-	-	447.3	223.7
K_2_SO_4_	871.3	435.7	-	-
KH_2_PO_4_	272.2	136.1	272.2	136.1
MgSO_4_·7H_2_O	985.9	493.0	985.9	493.0
CaCl_2_	1109.8	554.9	333.0	166.5

**Table 2:** Functional annotation of the ramie transcriptome in five public databases searched.

Database	Number of Unigenes	Percentage (%)
NCBI non-redundant protein sequences (Nr)	56,802	36.72
Swiss-Prot	35,660	23.05
Protein family (Pfam)	39,358	25.44
Gene Ontology (GO)	26,711	17.27
KEGG Orthology (KO)	11,912	7.7
Annotated in all databases	6,414	4.15
Annotated in at least one database	64,848	41.92
Total Unigenes	154,691	100

**Table 3:** DEG/DEP regulation patterns related to four major metabolic processes responding to water and N deficiency. The number before the slash “/” indicated the number of DEGs involved and the number behind indicate the number of DEPs involved.

Metabolic Processes	Regulation Trend	WNd vs. WdNd	WdN vs. WdNd	WdNd vs. CK	WNd vs. CK	WdN vs. CK
Photosynthesis-related	Up	3/16	0/14	0/12	0/5	1/9
	Down	1/4	4/15	2/24	0/11	21/20
Ethylene responding	Up	13/1	0/0	0/0	0/0	5/1
	Down	2/0	0/0	8/1	0/0	13/2
Glycolysis-related	Up	54/7	0/19	11/11	9/1	24/15
	Down	21/9	1/6	4/16	1/7	46/15
Nitrogen-related	Up	63/2	0/4	9/1	6/1	23/5
	Down	30/4	8/2	9/2	3/1	74/2

**Table 4:** Spearman coefficient of five different correlated types in comparison groups under different water and N conditions. NA indicates that no proteins/genes were detected in corresponding type or not enough data was available for calculating coefficient. The minus symbol before the numbers indicates negative correlation between DEPs and DEGs. DEPs/DEGs-SameTrend indicates same trend in DEPs and DEGs, such as upregulated DEPs simultaneously with upregulated DEGs, or downregulated DEPs simultaneously with downregulated DEGs. DEPs/DEGs-Opposite indicates opposite trend in DEPs and DEGs, such as upregulated DEPs simultaneously with downregulated DEGs and vice versa. DEPs/NDEGs indicates that there were DEPs but non-correlated DEGs. NDEPs/DEGs indicates that there are DEGs but non-correlated DEPs. NDEPs/NDEGs indicates that there are none DEPs or DEGs. NDEPs and NDEGs indicate non-differentially expressed proteins and non-differentially expressed genes, respectively.

Comparisons	WNd vs. WdNd	WdN vs. WdNd	WdNd vs. CK	WNd vs. CK	WdN vs. CK
DEPs/DEGs-SameTrend	0.5046	NA	−0.6018	NA	0.5542
DEPs/DEGs-Opposite	NA	NA	NA	NA	−0.2571
DEPs/NDEGs	0.4773	0.0186	0.4028	0.1212	0.2402
NDEPs/DEGs	0.3743	1	0.2913	0.1133	0.2989
NDEPs/NDEGs	0.2255	−0.0666	0.2371	−0.0231	0.1198

**Table 5:** KEGG pathways (corrected *P* value < 0.05) for the DEGs/DEPs by association analysis of transcriptome and proteome.

Comparisons	KEGG Pathways	Number of Proteins	Number of Genes
WNd vs. WdNd	Amino sugar and nucleotide sugar metabolism	8	16
	Pentose and glucuronate interconversions	5	8
	Metabolism of xenobiotics by cytochrome P450	3	7
	Drug metabolism—cytochrome P450	3	7
WdN vs. CK	Phenylpropanoid biosynthesis	8	10
	Photosynthesis—antenna proteins	5	10
WdNd vs. CK	Pentose and glucuronate interconversions	9	3

## Supplementary Materials

The following are available online at https://www.mdpi.com/2223-7747/9/10/1267/s1, Figure S1: Functional annotation of ramie transcriptome against GO database, Figure S2: Functional annotation of ramie transcriptome against KEGG database, Figure S3: Cluster analysis of DEGs and DEPs in WNd vs. WdNd, Figure S4: Cluster analysis of DEGs and DEPs in WdNd vs. CK, Figure S5: Cluster analysis of DEGs and DEPs in WdN vs. CK; Table S1: Details of all identified proteins, Table S2: DEGs involved in photosythesis, ethylene responding, glycolysis and nitrogen metabolism, Methods S-1: RNA-Seq libraries construction, sequencing and bioinformation analysis, Method S-2. Protein sample preparation and LC-MS/MS Analysis.
